# ﻿A new species of the *Cyrtodactyluschauquangensis* group (Squamata, Gekkonidae) from the borderlands of extreme northern Thailand

**DOI:** 10.3897/zookeys.1203.122758

**Published:** 2024-05-30

**Authors:** L. Lee Grismer, Anchalee Aowphol, Jesse L. Grismer, Akrachai Aksornneam, Evan S. H. Quah, Matthew L. Murdoch, Jeren J. Gregory, Eddie Nguyen, Amanda Kaatz, Henrik Bringsøe, Attapol Rujirawan

**Affiliations:** 1 Herpetology Laboratory, Department of Biology, La Sierra University, 4500 Riverwalk Parkway, Riverside, California 92505, USA La Sierra University Riverside United States of America; 2 Department of Herpetology, San Diego Natural History Museum, PO Box 121390, San Diego, California, 92112, USA Universiti Malaysia Sabah Kota Kinabalu Malaysia; 3 Institute for Tropical Biology and Conservation, Universiti Malaysia Sabah, Jalan UMS, 88400, Kota Kinabalu, Malaysia San Diego Natural History Museum San Diego United States of America; 4 Animal Systematics and Ecology Speciality Research Unit, Department of Zoology, Faculty of Science, Kasetsart University, Bangkok 10900, Thailand Kasetsart University Bangkok Thailand; 5 Biodiversity Center, Kasetsart University, Bangkok 10900, Thailand National University of Singapore Singapore Singapore; 6 Lee Kong Chian Natural History Museum, National University of Singapore, 2 Conservatory Drive, 117377, Singapore Universiti Sains Malaysia Penang Malaysia; 7 School of Biological Sciences, Universiti Sains Malaysia, 11800 Minden, Penang, Malaysia La Sierra University California United States of America; 8 Irisvej 8, DK-4600 Køge, Denmark Unaffiliated Køge Denmark

**Keywords:** Bent-toed gecko, genetics, Indochina, integrative taxonomy, karst, morphology

## Abstract

Phylogenetic and morphological analyses delimit and diagnose, respectively, a new population of a karst-dwelling *Cyrtodactylus* from extreme northern Thailand. The new species, *Cyrtodactylusphamiensis***sp. nov.**, of the *chauquangensis* group inhabits karst caves and outcroppings and karst vegetation in the vicinity of Pha Mi Village in Chiang Rai Province, Thailand. Within the *chauquangensis* group, *Cyrtodactylusphamiensis***sp. nov.** is the earliest diverging species of a strongly supported clade composed of the granite-dwelling *C.doisuthep* and the karst-dwelling sister species *Cyrtodactylus* sp. 6 and *C.erythrops*. The nearly continuous karstic habitat between the type locality of *Cyrtodactylusphamiensis***sp. nov.** and its close relatives *Cyrtodactylus* sp. 6 and *C.erythrops*, extends for approximately 200 km along the border region of Thailand and the eastern limit of the Shan Plateau of Myanmar. Further exploration of this region, especially the entire eastern ~ 95% of the Shan Plateau, will undoubtably recover new populations whose species status will need evaluation. As in all other countries of Indochina and northern Sundaland, the continual discovery of new karst-dwelling populations of *Cyrtodactylus* shows no signs of tapering off, even in relatively well-collected areas. This only highlights the conservation priority that these unique karstic landscapes still lack on a large scale across all of Asia.

## ﻿Introduction

**Figure 1.**
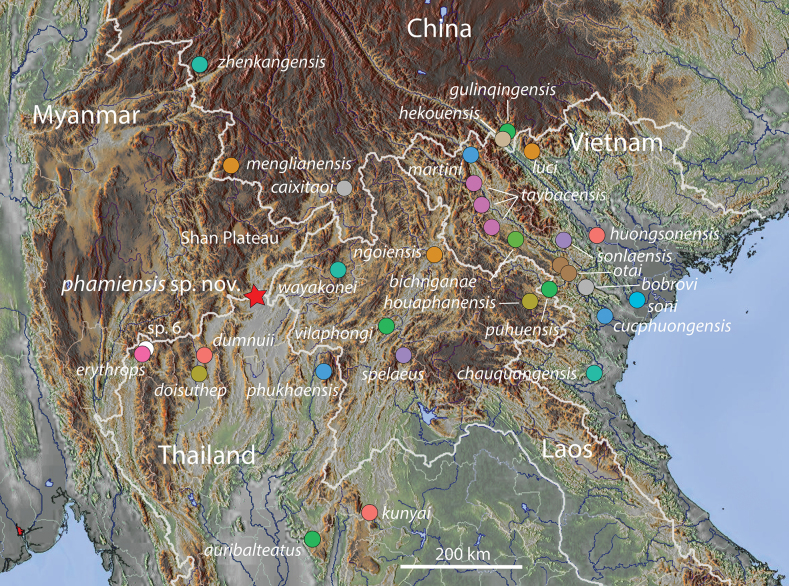
Distribution of nominal species and unnamed populations of the *Cyrtodactyluschauquangensis* group. The star denotes the type locality of *Cyrtodactylusphamiensis* sp. nov.

The borderlands of northwestern Thailand, western Laos, and south-central China encompass some of the most complex topography of Indochina. Rugged mountain ranges interleaved by deep gorges; wide, arid basins, and major river drainages, merge imperceptibly with those of the eastern uplands of Myanmar’s Shan Plateau. Although many species of *Cyrtodactylus* occupy the karstic borderlands girdling the Shan Plateau, none are yet known to occur within its rugged eastern topography. This collecting artifact is most noticeable in the distribution of the *chauquangensis* group (sec. Grismer et al. 2021) where the eastern border of Myanmar encloses a large unoccupied wedge in the western section of this group’s overall range (Fig. 1). The majority of the 28 nominal species of this group inhabit a fairly continuous karstic landscape that stretches from northwestern Thailand and south-central China, eastward through northern Laos to northwestern Vietnam west of the Red River, the exception being *C.gulinqingensis* Liu, Li, Hou, Orlov & Ananjeva, 2021 from Yunnan Province of southern China and *C.luci* Tran, Do, Pham, Phan, Ngo, Le, Ziegler & Nguyen, 2024 from Lao Cai Province in northern Vietnam which lie on the eastern edge of the Red River (Liu et al. 2021, 2023; Tran et al. 2024). Except for *C.taybacensis* Pham, Le, Ngo, Ziegler & Nguyen, 2019 and *C.otai* Nguyen, Le, Pham, Ngo, Hoang, Pham & Ziegler, 2015 of Vietnam (Nguyen et al. 2015a, 2017; Pham et al. 2019), most species in the *chauquangensis* group are known only from their type localities, underscoring the specialized, restrictive life history of karst-dwelling species coupled to the generally fragmented nature of karstic landscapes.

While conducting fieldwork during March of 2023 in the district of Mae Sai along the Thai-Myanmar border in extreme northern Chiang Rai Province, Thailand, we discovered a new population of karst-dwelling *Cyrtodactylus* near Pha Mi Village at the Wat Pa (= temple) Pha Mi as well as from adjacent areas within the same karstic range. Molecular phylogenetic analyses indicated this population was deeply embedded within the *chauquangensis* group and composed the sister species to a lineage containing three other species from northern Thailand. Based on this, and statistically significant diagnostic results from univariate and multivariate analyses, we hypothesize this new population constitutes a new species and as such, describe it below.

## ﻿Materials and methods

### ﻿Sampling

The gecko specimens were collected during a field survey at Pha Mi Village, Wiang Phang Kham Subdistrict, Mae Sai District, Chiang Rai Province, Thailand from 25–26 March 2023 (Fig. 1). Geographical coordinates with elevation of each specimen were collected using a Garmin GPSMAP 64^st^. After collection, the specimens were photographed in life prior to preservation. All specimens were then humanely euthanized using tricaine methanesulfonate (MS-222) solution (Simmons 2015). Liver tissue was immediately dissected from the euthanized specimens, preserved in 95% ethyl alcohol, and stored at -20 °C for genetic analysis. Voucher specimens were then initially fixed in 10% formalin and later transferred to 70% ethyl alcohol for long-term storage. The type series and tissue samples were deposited in the herpetological collection of the Zoological Museum, Kasetsart University, Bangkok, Thailand (**ZMKU**).

### ﻿Molecular data

Genomic DNA was isolated from liver or skeletal muscle samples stored in 95% ethanol using the Qiagen DNeasyTM tissue kit (Valencia, CA, USA). NADH dehydrogenase subunit 2 gene (ND2) and downstream tRNA-Trp, tRNA-Ala, and tRNA-Asn were chosen for phylogenetic analyses with 10 specimens newly sequenced for this work. ND2 was amplified using a double-stranded Polymerase Chain Reaction (PCR) under the following conditions: 2.5 μl genomic DNA (~10–30 ng), 2.5 μl light strand primer (5 μM), 2.5 μl heavy strand primer (5 μM), 1.0 μl dinucleotide pairs (1.0 μM), 2.0 μl 5× buffer (2.0 μM), 1.0 MgCl 10× buffer (1.0 μM), 0.18 μl Taq polymerase (5u/μl), and 9.8s μl ultrapure H2O at n + 1. PCR reactions were executed on a BIO RAD T-100 Thermal Cycler under the following conditions: initial denaturation at 94 °C for 4 min, followed by a second denaturation at 94 °C for 30 s, annealing at 52 °C for 30 s, followed by a cycle extension at 68 °C for 1:30 min repeated for 35 cycles, followed by a final extension cycle run at 68 °C for 7 min. All PCR products were visualized on a 1.0% agarose electrophoresis gel. Successful targeted PCR products were outsourced to GENEWIZ® for PCR purification, cycle sequencing, and sequencing. Primers used for amplification and sequencing are presented in Murdoch et al. (2019: table 2). Sequences were analyzed from both the 3' and the 5' ends separately to confirm congruence between the reads. Both the forward and the reverse sequences were uploaded and edited in GeneiousTM v. 5.5.6 (Drummond et al. 2011) and were edited therein. The protein-coding region of the ND2 sequence was aligned by eye. Mesquite v. 3.04 (Maddison and Maddison 2015) was used to calculate the correct amino acid reading frame and to confirm the lack of premature stop codons. The GenBank accession numbers for all specimens are in Suppl. material 1.

### ﻿Phylogenetic analyses

Three different partition schemes, codon, gene, and unpartitioned, were run for three model based phylogenetic analyses – Maximum Likelihood (ML), Bayesian Inference, (BI) and Bayesian Evolutionary Analysis Sampling Trees (BEAST) – in order to search for significant support for the weak nodes in recent analyses (Liu and Rao 2021a, 2021b, 2022; Chomdej et al. 2022) using NADH dehydrogenase subunit 2 (ND2) gene and its flanking tRNAs. *Cyrtodactylusdammathetensis* Grismer, Wood, Thura, Zin, Quah, Murdoch, Grismer, Lin, Kyaw & Lwin, 2017 and *C.sinyineensis* Grismer, Wood, Thura, Zin, Quah, Murdoch, Grismer, Lin, Kyaw & Lwin, 2017 (in Grismer et al. 2018a) were used as outgroups to root the ML and BI trees based on Grismer et al. (2021). Maximum Likelihood (ML) analyses were implemented using the IQ-TREE webserver (Nguyen et al. 2015b; Trifinopoulos et al. 2016). One-thousand bootstrap pseudoreplicates via the ultrafast bootstrap (UFB; Hoang et al. 2018) approximation algorithm were employed, and nodes having UFB values of 95 and above were considered strongly supported (Minh et al. 2013). We considered nodes with values of 90–94 as well-supported. After removing outgroup taxa, uncorrected pairwise sequence divergences were calculated in MEGA 11 (Tamura et al. 2021) using the pairwise deletion option to remove gaps and missing data from the alignment prior to calculation.

Bayesian inference (BI) analyses was implemented in MrBayes 3.2.3 on XSEDE (Ronquist et al. 2012) using CIPRES (Cyberinfrastructure for Phylogenetic Research; Miller et al. 2010). Two simultaneous runs were performed with four chains, three hot and one cold. The simulations ran for 50,000,000 generations, were sampled every 5,000 generations using a Markov Chain Monte Carlo (MCMC), and the first 10% of each run were discarded as burn-in. Stationarity and parameter files from each run were checked in Tracer v. 1.7 (Rambaut et al. 2018) to ensure effective sample sizes (ESS) were above 200 for all parameters. Nodes with Bayesian posterior probabilities (BPP) of 0.95 and above were considered strongly supported (Huelsenbeck et al. 2001; Wilcox et al. 2002). We considered nodes with values of 0.90–0.94 as well-supported.

Input files constructed in BEAUti (Bayesian Evolutionary Analysis Utility) v. 2.4.6 were run in BEAST (Bayesian Evolutionary Analysis Sampling Trees) v. 2.4.6 (Drummond et al. 2012) on CIPRES (Cyberinfrastructure for Phylogenetic Research; Miller et al. 2010) in order to generate BEAST phylogenies. An optimized relaxed clock with unlinked site models and linked tree and clock models were employed for each run. bModelTest (Bouckaert and Drummond 2017), implemented in BEAST, was used to numerically integrate over the uncertainty of the three input files while simultaneously estimating phylogeny using a Markov Chain Monte Carlo (MCMC). MCMC chains were run using a Yule prior for 50 million generations and logged every 5,000 generations. The BEAST log files were visualized in Tracer v. 1.7 (Rambaut et al. 2018) to ensure effective sample sizes (ESS) were well above 200 for all parameters. Maximum clade credibility trees using mean heights at the nodes were generated using TreeAnnotator v. 1.8.0 (Rambaut and Drummond 2013) with a burn-in of 10%. Nodes with Bayesian posterior probabilities (BPP) of 0.95 and above were considered strongly supported (Huelsenbeck et al. 2001; Wilcox et al. 2002). We considered nodes with values of 0.90–0.94 as well-supported.

### ﻿Species delimitation

The general lineage concept (GLC: de Queiroz 2007) adopted herein proposes that a species constitutes a population of organisms evolving independently from other such populations owing to a lack of gene flow. By “independently,” it is meant that new mutations arising in one species cannot spread readily into another species (Barraclough et al. 2003; de Queiroz 2007). Under the GLC implemented herein, molecular phylogenies were used to recover monophyletic mitochondrial lineages of individual(s) (i.e., populations) in order to develop initial species-level hypotheses, the grouping stage of Hillis (2019). Discrete color pattern data and morphological data were used to search for unique characters and patterns and compare their consistency with the previous species-level hypotheses designations, the construction of boundaries representing the hypothesis-testing step of Hillis (2019), thus providing lineage diagnoses independent of the molecular analyses. In this way, delimiting (phylogeny) and diagnosing (taxonomy) species are not conflated (Frost and Hillis 1990; Frost and Kluge 1994; Hillis 2019).

### ﻿Morphological data

Morphological data included morphometric, meristic, and categorical morphological and color pattern characters. Measurements were taken on the left side of the body to the nearest 0.1 mm using Mitutoyo dial calipers under a Nikon SMZ 1500 dissecting microscope and follow Grismer and Grismer (2017) and Grismer et al. (2018a). Measurements taken were: snout-vent length (**SVL**), taken from the tip of the snout to the vent; tail length (**TL**), taken from the vent to the tip of the tail; tail width (**TW**), taken at the base of the tail immediately posterior to the postcloacal swelling; forearm length (**FL**), taken on the ventral surface from the posterior margin of the elbow while flexed 90° to the inflection of the flexed wrist; tibia length (**TBL**), taken on the ventral surface from the posterior surface of the knee while flexed 90° to the base of the heel; axilla to groin length (**AG**), taken from the posterior margin of the forelimb at its insertion point on the body to the anterior margin of the hind limb at its insertion point on the body; head length (**HL**), the distance from the posterior margin of the retroarticular process of the lower jaw to the tip of the snout; head width (**HW**), measured at the angle of the jaws; head depth (**HD**), the maximum height of head measured from the occiput to base of the lower jaw; eye diameter (**ED**), the greatest horizontal diameter of the eye-ball; eye to ear distance (**EE**), measured from the anterior edge of the ear opening to the posterior edge of the bony orbit; snout length (**ES**), measured from anteriormost margin of the bony orbit to the tip of snout; eye to nostril distance (**EN**), measured from the anterior margin of the bony orbit to the posterior margin of the external naris; interorbital distance (**IO**), measured between the anterior-most edges of the bony orbits; ear length (**EL**), measured as the greatest vertical distance of the ear opening; and internarial distance (**IN**), measured between the nares across the rostrum.

Meristic characters evaluated were the number of supralabial scales (**SL**) counted from the largest scale immediately below the eyeball to the rostral scale; infralabial scales (**IL**) counted from the mental to the termination of enlarged scales just after the upturn of the mouth; the number of paravertebral tubercles (**PVT**) between limb insertions counted in a straight line immediately left or right of the vertebral column; the number of longitudinal rows of body tubercles (**LRT**) counted transversely across the center of the dorsum from one ventrolateral fold to the other; the number of longitudinal rows of ventral scales (**VS**) counted transversely across the center of the abdomen from one ventrolateral fold to the other; the number of expanded subdigital lamellae on the fourth toe (**E4TL**) counted from the base of the first phalanx to the large scale on the digital inflection; the number of unexpanded subdigital lamellae on the fourth toe (**U4TL**) counted from the digital inflection to the end of the digit and including the claw sheath; the total number of expanded subdigital lamellae on the fourth toe (**T4TL** = E4TL+U4TL) counted from the base of the first phalanx where it contacts the body of the foot to the claw and including the claw sheath (see Murdoch et al. 2019: fig. 2); and the total number of enlarged femoral scales (**FS**) from each thigh combined as a single metric. In some species, only the distalmost FS are greatly enlarged, and the proximal scales are smaller whereas in others, the enlarged scales are continuous with the enlarged precloacal scales. The separation of the two scales rows was determined to be at a point even with the lateral body margin (see Murdoch et al. 2019: fig. 3). The number of enlarged precloacal scales (**PS**); the number of precloacal pores (**PP**) in males; the total number of femoral pores in males **(FP)**; the number of rows of enlarged post-precloacal scales (**PPS**) on the midline between the enlarged precloacal scales and the granular scales anterior to the vent; the number of postcloacal tubercles (**PCT**); the number of dark body bands (**BB**) between the nuchal loop (the dark band running from eye to eye across the nape) and the hind limb insertions; and numbers of light-colored (**LCB**) and dark-colored (**DCB**) caudal bands.

**Figure 2.**
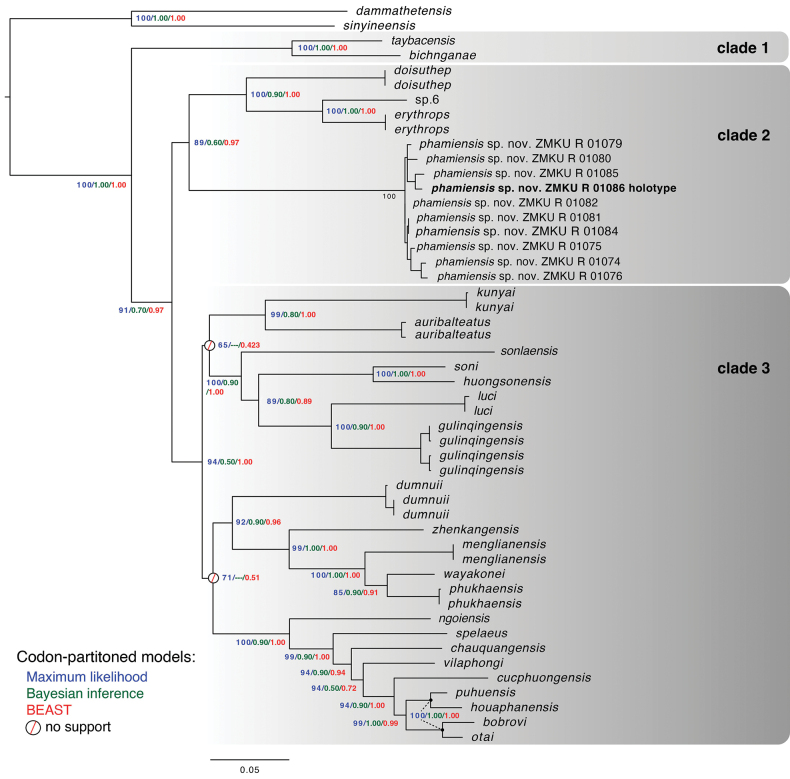
Maximum Likelihood topology of the *Cyrtodactyluschauquangensis* group with nodal support from the three best performing ML, BI, and BEAST analyses.

**Figure 3.**
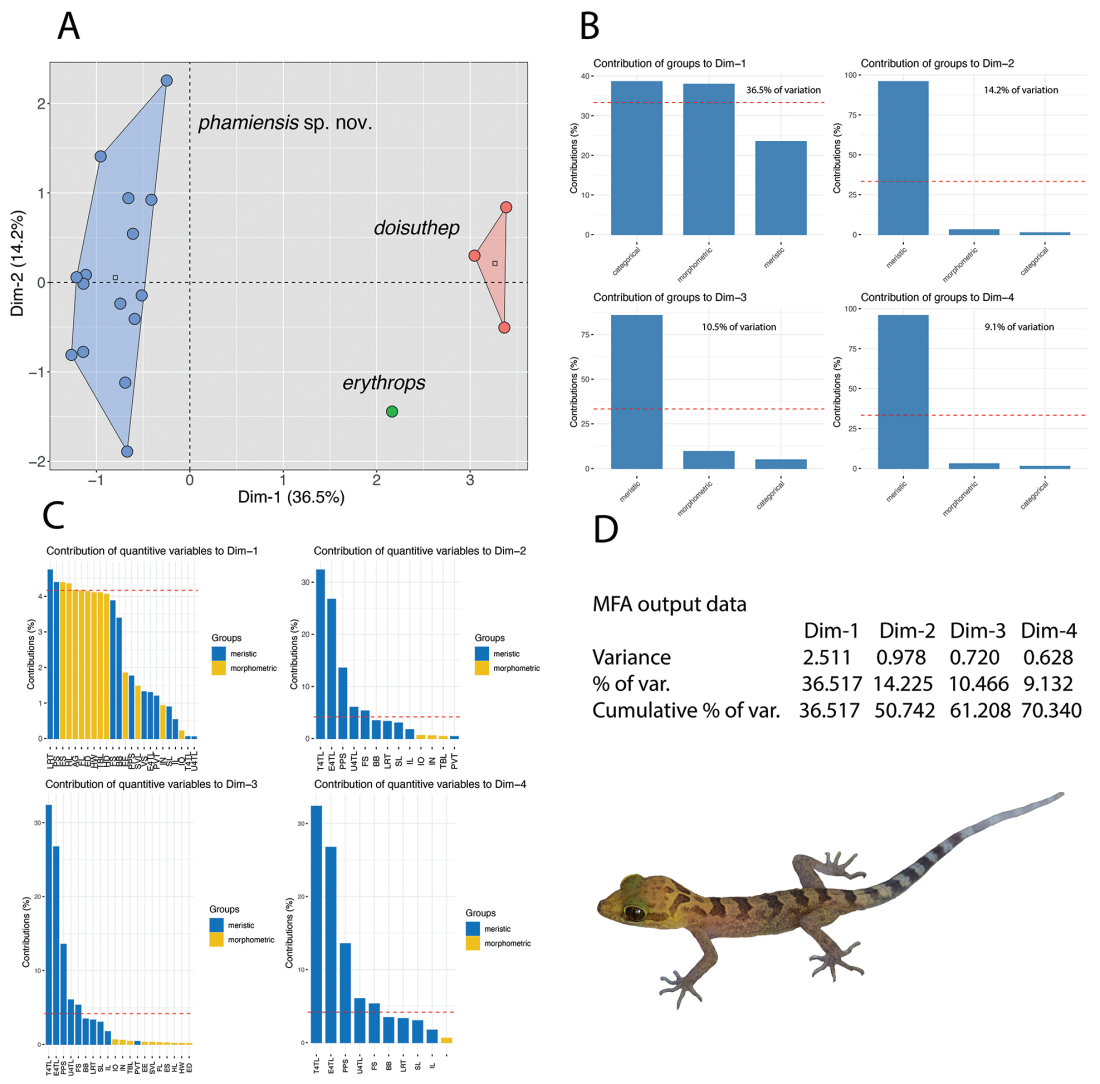
**A**MFA of the species of clade 2 (Fig. 2) **B** Percent contributions of each data type to the inertia of dimensions 1–4 of the MFA. Percentage values on the bar graphs are the amounts of inertia for their respective dimensions **C** Percent contribution of the quantitative variables to the dimensions 1–4 of the MFA. Dotted red line is the mean percentage if all values were equal **D**MFA output data showing the variance, percent variance, and the cumulative percent of 70.3% of the total variance for dimensions 1–4.

Categorical morphological and color pattern characters examined were tubercles extending beyond base of tail or not; femoral pores restricted to distal scales or not; body tubercles low, weakly keeled or raised, moderately to strongly keeled; enlarged femoral and precloacal scales continuous or not; pore-bearing femoral and precloacal scales continuous or not; enlarged proximal femoral scales ~ 1/2 size of distal femorals or not; medial subcaudals two or three times wider than long or not; medial subcaudals extend upward onto lateral surface of tail or not; nuchal loop divided medially or continuous; color of head in hatchings yellow or not (**HeadCol**); two posterior projections from nuchal loop present or not; nuchal loop with anterior azygous notch or not; triangular marking anterior to nuchal loop; posterior border of nuchal loop projected or smooth; band on nape present or absent; dorsal banding with paravertebral elements or not; dorsal body bands wider than interspaces or not (**IntSpac**); dorsal body bands with lightened centers or not; dorsal bands edged with white tubercles or not; dorsal tubercles brightly colored or dull (**BodTub**); dorsal bands straight or jagged; dark markings in dorsal interspaces or not; ventrolateral fold whitish or not; top of head diffusely mottled, blotched, or patternless light-colored reticulum on top of head or not (**HeadRetic**); anterodorsal margin of thighs darkly pigmented or not; anterodorsal margin of brachia darkly pigmented or not; white caudal bands with dark markings or not; white caudal bands encircle tail or not; dark caudal bands wider than light caudal bands or not; and mature regenerated tail spotted. HeadCol, IntSpac, BodTub, and HeadRetic were used in the multiple factor analysis (MFA) see below because they could be consistently coded across other taxa.

### ﻿Statistical analyses

All statistical analyses were conducted using R Core Team (2021). Low sample sizes and incomplete character data throughout species of the *chauquangensis* group, precluded meaningful group-wide univariate analyses. As the next best alternative, we employed a MFA using the R package *FactorMineR* (Husson et al. 2017) and visualized it using the *Factoextra* package (Kassambara and Mundt 2017) for a clade of species from northwestern Thailand to which the Pha Mi population belongs (see below). In so doing, we were able to statistically defend the morphospatial placement of the Pha Mi population as significant (see below). We believe this is superior to the majority of current species diagnoses that provide no statistical analyses and relay only numeric ranges with little to no regard to sample size. The MFA was conducted on a concatenated data set comprised of 12 meristic (SL, IL, PVT, LRT, VS, E4TL, U4TL, T4TL, PS, PPS, FS, and BB), 12 morphometric (SVL, FL, TBL, AG, HL, HW, HD, ED, EE, ES, IO, and IN), and four categorical (HeadCol, IntSpac, BodTub, and HeadRetic) characters. To remove potential effects of allometry in the morphometric characters, size was normalized using the following equation: X_adj_ = log(X)-β[log(SVL)-log(SVL_mean_)], where X_adj_ = adjusted value; X = measured value; β = unstandardized regression coefficient for each population; and SVL_mean_ = overall average SVL of all populations (Thorpe 1975, 1983; Turan 1999; Lleonart et al. 2000). The morphometrics of each species were normalized separately and then concatenated so as not to conflate intra- with interspecific variation (Reist 1986). All data were scaled to their standard deviation to insure they were analyzed on the basis of correlation and not covariance. MFA is a global, unsupervised, multivariate analysis that incorporates qualitative and quantitative data (Pagès 2015) simultaneously, making it possible to analyze different data types in a nearly total morphological evidence environment. In an MFA, each individual is described by a different set of variables (i.e., characters) which are structured into different data groups in a global data frame, in this case, quantitative data (i.e., meristics and normalized morphometrics) and categorical data (i.e. color pattern characters). In the first phase of the analysis, separate multivariate analyses are carried out for each set of variables: principal component analyses (PCA) for each quantitative data set and a multiple correspondence analysis (MCA) for the categorical data. The data sets are then normalized separately by dividing all their elements by the square root of their first eigenvalue. For the second phase of the analysis, these normalized data sets are concatenated into a single matrix for a final global PCA of the normalized data. Standardizing the data in this manner prevents one data type from overleveraging another. In other words, the normalization of the data in the first phase prevents data types with the greatest number of characters or the greatest amount of variation from outweighing other data types in the second phase. This way, the contributions of each data type to the overall variation in the data set is scaled to define the morphospatial distance between individuals as well as calculating each data type’s contribution to the overall variation in the analysis (Pagès 2015; Kassambara and Mundt 2017).

A non-parametric permutation multivariate analysis of variance (PERMANOVA) from the *vegan* package 2.5–3 in R (Oksanen et al. 2020) was used to determine if the centroid locations and group clusters of each species/population were statistically different from one another (Skalski et al. 2018) based on the MFA load scores of dimensions 1–5. Using loading scores as opposed to raw data, allows for the incorporation of the categorical characters which cannot be run in a PERMANOVA untransformed. The analysis calculates a Euclidean (dis)similarity matrix using 50,000 permutations. A pairwise *post hoc* test calculates the differences between the populations, generating a Bonferroni-adjusted *p* value and a pseudo-*F* ratio (*F* statistic). A *p* < 0.05 is considered significant and larger *F* statistics indicate more pronounced group separation. A rejection of the null hypothesis (i.e., centroid positions and the spread of the data points [i.e., clusters] are no different from random) signifies a statistically significant difference between species/populations.

*T*-tests were run for each character between the Pha Mi population (*n* = 15) and *Cyrtodactylusdoisuthep* Kunya, Panmongkol, Pauwels, Sumontha, Meewasana, Bunkhwamdi & Dangsri, 2014 (*n* = 3) to ascertain which means of the numeric characters differed significantly (*p* < 0.05). *F*-tests were run *a priori* to test for homogeneity of variances. If the variances were homogeneous (*p* ≥ 0.05), a Student two sample *t*-test was employed. If the variances were not homogeneous (*p* < 0.05), a Welch two sample *t*-test was employed. Both tests employed a Bonferroni correction factor to calculate an adjusted *p*-value. *Cyrtodactylusdoisuthep* was chosen for comparison because it was the only species in clade 2 (see below) that had more than two samples. *Cyrtodactyluserythrops* Bauer, Kunya, Sumontha, Niyomwan, Panitvong, Pauwels, Chanhome & Kunya, 2009 had an *n* = 1 and no data exist for *Cyrtodactylus* sp. 6 (Chomdej et al. 2021). For comparisons with all other species of the *chauquangensis* group, we put together the most complete dataset possible following Pham et al. (2019) and supplemented it with the original descriptions of recently described species (Schneider et al. 2020; Liu and Rao 2021b, 2022; Liu et al. 2021, 2023; Zhang et al. 2021; Chomdej et al. 2022).

## ﻿Results

No competing topological differences were recovered among nine phylogenies and the codon-partitioned data performed best among the three models (i.e., ML, BI, and BEAST) based on likelihood scores (Table 1). Nodal support among the models differed across the trees and codon-partitioned BI data recovered two polytomies (Fig. 2). Substitution models for the codon-partitioned ML tree based on the Bayesian Information Criterion (BIC) in ModelFinder (Kalyaanamoorthy et al. 2017) selected HKY+F+I+G4 codon position 1, TPM2+F+G4 for position 2, TPM3+F+G4 for position 3, and TIMe+G4 the non-coding RNAs. bModel test was used to co-estimate the site models and the phylogenies simultaneously for the BI and BEAST analyses.

Of the three best performing codon-partitioned phylogenies, the BEAST analysis performed best in that it recovered the greatest number of strongly supported ingroup nodes (20) and the fewest number of moderately and unsupported nodes (Table 1, Fig. 2). Two nodes had no support from any of the nine analyses and one other node was very close to being moderately supported in the ML and BEAST analyses (UFB = 89, BEASTBPP = 0.89). The monophyly of the *chauquangensis* group was strongly supported in all analyses. The ML and BEAST analyses recovered three major clades within the *chauquangensis* group, only one of which (clade 1) was strongly supported in all analyses (100, 1.00, 1.00; ML UFB, BIBPP, and BEASTBPP, respectively and throughout). The earliest diverging clade 1, contains *Cyrtodactylusbichnganae* Ngo & Grismer, 2010 and *C.taybacensis* from the Hoang Lien Son Mountain Range in northwestern Vietnam. Clade 2, strongly supported only in the BEAST analysis (89, 0.60, 0.97), contains *C.doisuthep*, *C.erythrops*, *Cyrtodactylus* sp. 6, and the Pha Mi population – the latter recovered as the sister taxon to the remaining species. The sister relationship between clades 2 and 3 was strongly supported only in the ML and BEAST analyses (91, 0.70, 0.97). The monophyly of clade 3, variably supported (94, 0.50, 1.00), contains the remaining 21 species that collectively range from the borderlands of western Yunnan, eastward across northern Thailand and Laos and into northwestern Vietnam. Clade 3 is a polytomy in that no analysis offered any support for the basal nodes. Varying support from all three analyses recovered at least four major lineages withing clade 3. Although the same equivocation was found in the most recent analyses of this group (Liu and Rao 2021a, 2021b, 2022; Liu et al. 2023; Chomdej et al. 2022; Tran et al. 2024), it went unnoted. The mean uncorrected pairwise sequence divergence between the Pha Mi population and the species of clade 2 ranged from 13.48–14.49%. Genetic distances within the Pha Mi population ranged from 0.00–1.81% and distances between the Pha Mi population and the remaining species of the *chauquangensis* group was 13.72–17.34% (Suppl. material 2).

The MFA analysis recovered all three nominal species of the clade 2 to be widely separated from one another along dimension 1 which accounted for 36.5% of the variation in the data set (Fig. 3A, D). Both *Cyrtodactyluserythrops* and *C.doisuthep* were separated from one another along dimension 2, accounting for an additional 14.2% of the variation, but were not separated from the Pha Mi population (Fig. 3A). Categorical and morphometric characters contributed most of the variation along dimension 1 and meristc data contributed to the majority of the variation along dimension 2 (Fig. 3B). Meristic characters LRT, PS followed by morphometric characters ES, HL, AG, FLEDHW, TBL and HD contributed to the majority of the variation along dimension 1 (Fig. 3C). Meristic characters T4TL, and E4TL contributed to the majority of the variation along dimension 2. The PERMANOVA analysis indicated that the Pha Mi population differed significantly in morphospace from *C.doisuthep* but not from *C.erythrops* (Table 2). The latter case owes to the sample size of *C.erythrops* (*n* = 1) which precluded its statistically significant differentiation from *C.doisuthep* as well, even though it was widely separated from both species.

The results of the *t*-tests (Table 3) mirrored those of the PERMANOVA in that the Pha Mi population differed significantly from *Cyrtodactylusdoisuthep* in having fewer supralabials (SL) and enlarged femorals (FS); more precloacals (PS); a shorter axilla-groin length (AG); shorter forelimbs (FL) and tibias (TBL); a shorter, wider, and flatter head (HL, HW, HD, respectively) with a shorter snout (ES) and postorbital region (EE), a smaller eyeball (ED); and a nearly significantly different smaller snout-vent length (SVL). Non-statistical comparisons of a range of other selected characters illustrates how the Pha Mi population may differ from other species in the *chauquangensis* group (Table 4). Raw data would bear these differences out more clearly but were unavailable to us.

### ﻿Taxonomy

**Figure 4.**
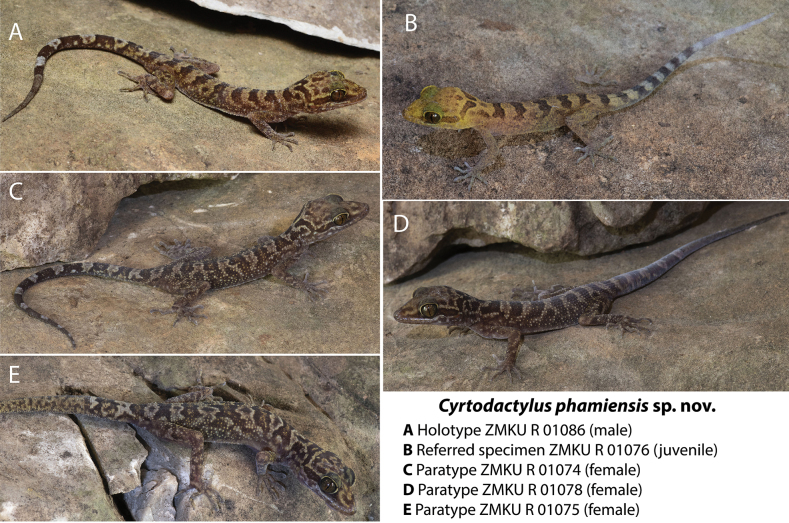
Selected individuals of the type series and referred specimen of *Cyrtodactylusphamiensis* sp. nov. from Pha Mi Village, Wiang Phang Kham Subdistrict, Mae Sai District, Chiang Rai Province, Thailand.

Given that the Pha Mi population is not phylogenetically embedded within any other species of the *chauquangensis* group nor is it sister to any other species (Fig. 2); bears a large, uncorrected pairwise sequence divergence of 13.5–14.5% from its closest relatives in clade 2, is morphospatially isolated form all other species in clade 2 along the ordination of dimensions 1 and 2 (Fig. 3) and occupies a significantly unique position with respect to *Cyrtodactylusdoisuthep* (Table 2); has significantly different mean values from those of *C.doisuthep* in three meristic and 10 adjusted morphometric characters (Table 3); differs from *C.doisuthep* and *C.erythrops* in lacking a light-colored reticulum on the top of the head; having hatchlings with yellow as opposed to tan colored heads (Fig. 4B); and a range of other potentially different character states from species in clades 1 and 3 (Table 4), we consider the most parsimonious hypothesis based these independent data sets to be that the Pha Mi population is a distinct species.

#### ﻿ Cyrtodactylus phamiensis sp. nov.

Taxon classificationAnimaliaSquamataGekkonidae

B98A591B-0227-5EF3-9844-5EB55624C364

https://zoobank.org/DDADBD8A-5234-4183-89A7-C5A3C015456A

**Figure 5.**
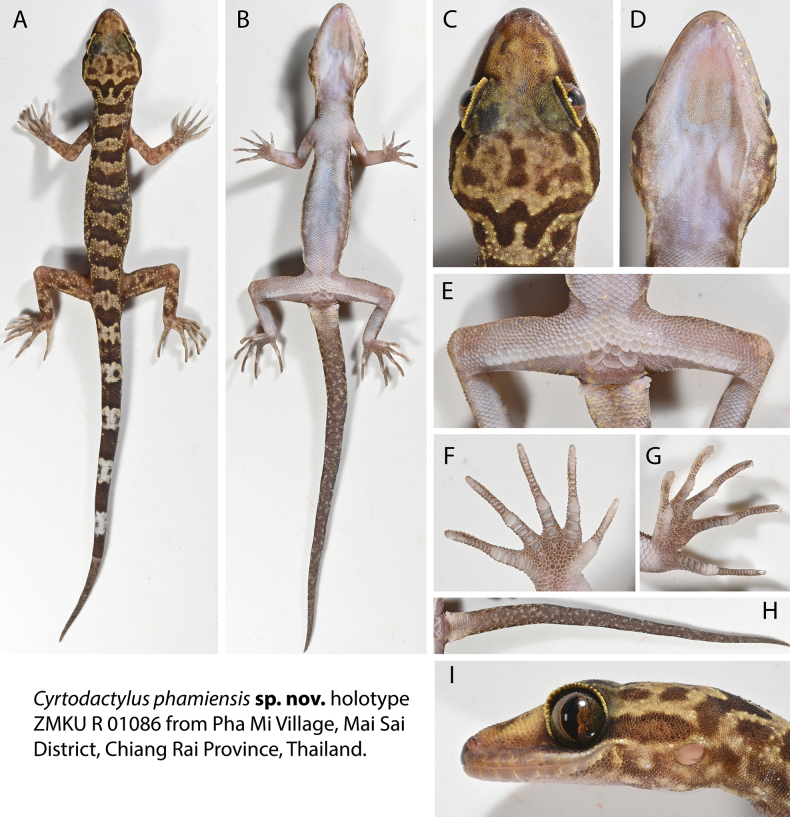
Adult male holotype of *Cyrtodactylusphamiensis* sp. nov. **A** dorsal view **B** ventral view **C** dorsal view of head **D** gular region **E** thighs and precloacal region **F** ventral view of right manus **G** ventral view of left pes **H** subcaudal region **I** lateral view of left side of head. Photographs by Attapol Rujirawan.

**Figure 6.**
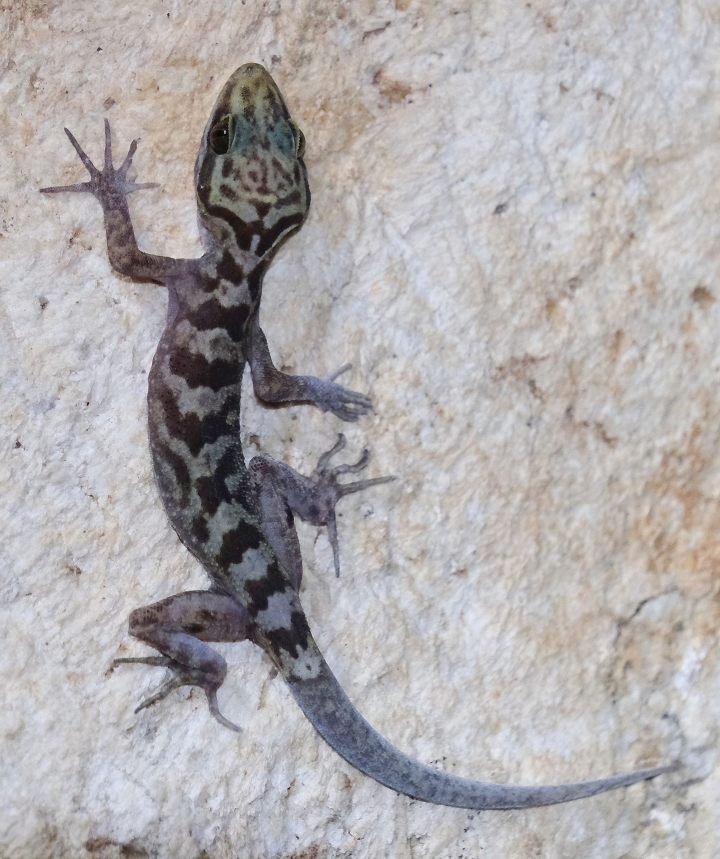
Cyrtodactyluscf.phamiensis sp. nov. from 5 km north of the type locality in Tham Pha Chom. Photograph by Ada Chornelia.

**Figure 7.**
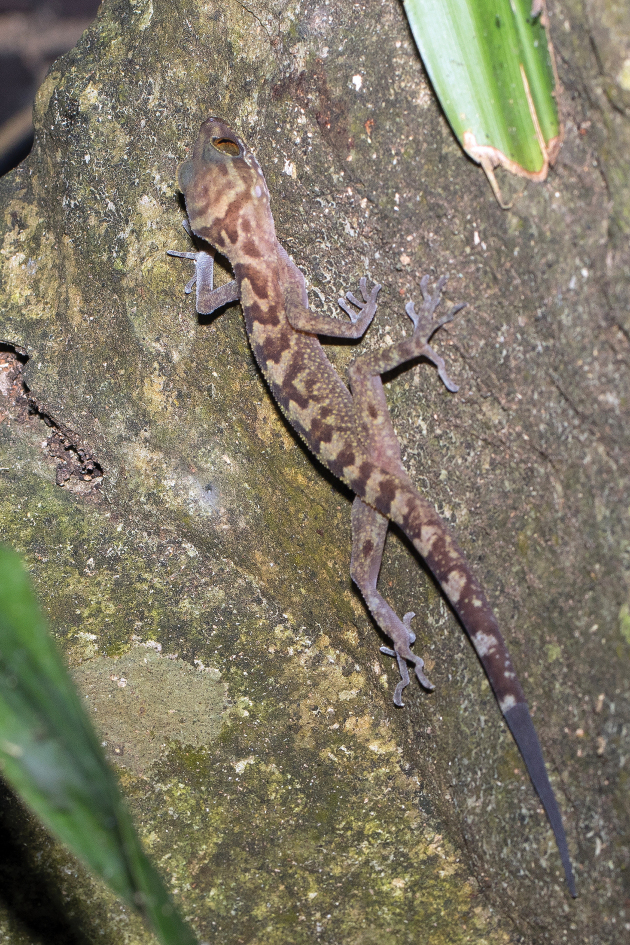
*Cyrtodactylusphamiensis* sp. nov. from 50 meters west-southwest of the type locality, immediately east of Pha Mi Village. 17 December 2022. Photograph by Henrik Bringsøe.

**Figure 8.**
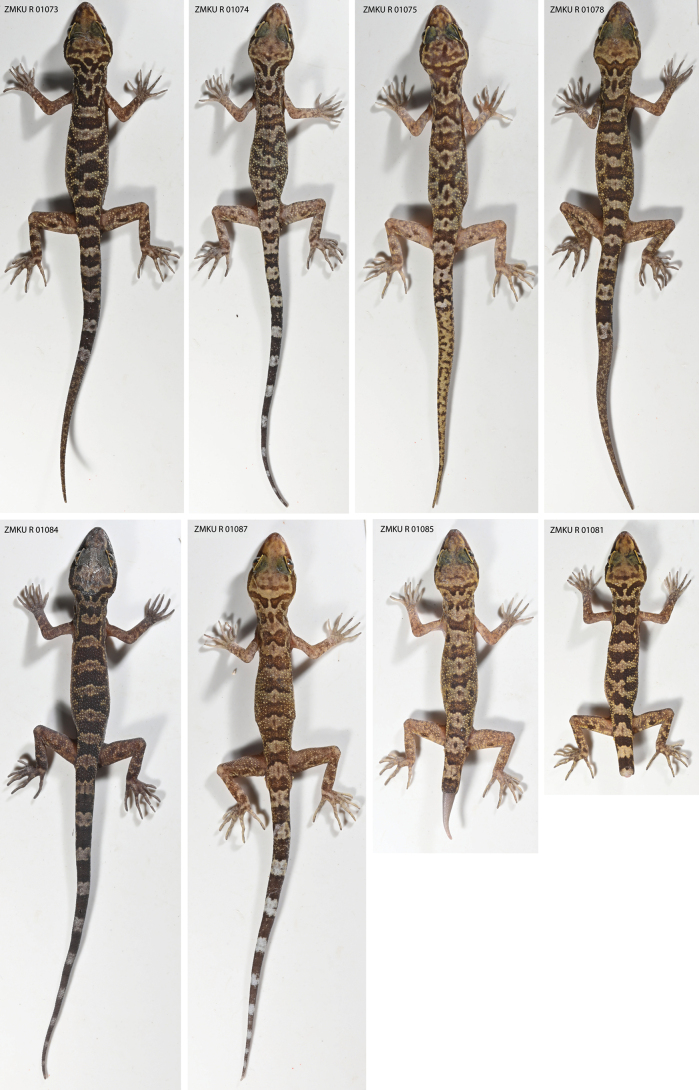
Paratypes of *Cyrtodactylusphamiensis* sp. nov. from Pha Mi Village, Wiang Phang Kham Subdistrict, Mae Sai District, Chiang Rai Province, Thailand. Photographs by Attapol Rujirawan.

Figs 4 , 5 , 6 , 7 , 8

##### Type material.

***Holotype*.** Adult male (ZMKU R 01086) collected from Pha Mi Village, Wiang Phang Kham Subdistrict, Mae Sai District, Chiang Rai Province, Thailand (20.40134°N, 99.85369°E; elevation 517 m a.s.l.) on 26 March 2023 by A. Aowphol, A. Rujirawan, A. Aksornneam, L.L. Grismer, J.L. Grismer, E.S.H. Quah, and M.L. Murdoch.

***Paratypes*.** Two adult males (ZMKU R 01085, ZMKU R 01087) and one adult female (ZMKU R 01084) bear the same collection data as the holotype. Four adult females (ZMKU R 01073–01075, ZMKU R 01078) and one adult male (ZMKU R 01081) bear the same collection data as the holotype except collected on 25 March 2023.

##### Referred specimens.

Six hatchlings. ZMKU R 01076–01077, ZMKU R 01079–01080 bear the same collection data as the holotype except were collected on 25 March 2023. ZMKU R 01082–01083 bear the same collection data as the holotype except collected from 20.39800°N, 99.85466°E; elevation 505 m a.s.l., on 25 March 2023.

##### Diagnosis.

*Cyrtodactylusphamiensis* sp. nov. can be separated from all other species of the *chauquangensis* group by the combination of having a maximum SVL = 74.4 mm (female); 8–12 supralabials; 9–11 infralabials; 30–43 paravertebral tubercles; 19–25 rows of longitudinally arranged tubercles; 29–37 longitudinal rows of ventrals; 6–9 expanded subdigital lamellae on the fourth toe; 12–14 unmodified subdigital lamellae on the fourth toe; 19–22 total subdigital lamellae on the fourth toe; 19–28 total number of enlarged femoral scales; 9–14 total number of femoral pores in males (*n* = 4); 6–11 enlarged precloacals; 4–6 precloacal pores in males (*n* = 4); two or three rows of large post-precloacal scales; enlarged femorals and enlarged precloacals continuous; proximal femorals usually smaller than distal femorals; femoral pores restricted to distal scales; body tubercles weakly keeled; small tubercles on forelimbs; tubercles extend beyond base of tail; medial subcaudals 2–3 times wider than long but not extending onto lateral surface of tail; nuchal loop often divided medially, bearing two posteriorly directed projections, no anterior azygous notch, projecting posterior margin; usually no triangular marking anterior to nuchal loop; dark-colored band on nape variably present; dark-colored dorsal bands lack paravertebral elements, have variably lightened centers, are edged with white tubercles, usually jagged in shape, and the same width or wider than interspaces; dark-colored markings in dorsal interspaces; no whitish ventrolateral fold; top of head in adults diffusely mottled, blotched; no light-colored reticulum on top of head; 4–6 dark-colored transverse body bands; 10–13 light-colored caudal bands on an original tail bearing dark-colored markings and not encircling tail (*n* = 7); 9–12 dark-colored caudal bands on an original tail and wider than light-colored caudal bands (*n* = 7); and mature regenerated tail mottled (*n* = 3) (Table 4).

##### Description of holotype.

(Figs 4A, 5, Suppl. material 3) Adult male SVL 68.5 mm; head moderate in length (HL/SVL 0.28), width (HW/HL 0.72), flattened (HD/HL 0.40), distinct from neck, triangular in dorsal profile; lores weakly concave anteriorly, weakly inflated posteriorly; prefrontal region concave; canthus rostralis rounded; snout elongate (ES/HL 0.40), flat, rounded in dorsal profile; eye large (ED/HL 0.31); ear opening elliptical, obliquely oriented, moderate in size; eye to ear distance slightly greater than diameter of eye; rostral rectangular, partially divided dorsally by inverted Y-shaped furrow, bordered posteriorly by large left and right supranasals, bordered laterally by first supralabials; external nares bordered anteriorly by rostral, dorsally by large supranasal, posteriorly by two moderately sized postnasals, bordered ventrally by first supralabial; nine (R, L) rectangular supralabials tapering abruptly to below midpoint of eye, first–fifth supralabials largest; 11 (R, L) infralabials tapering smoothly to slightly past the termination of enlarged supralabials to corner of mouth; scales of rostrum and lores flat, larger than granular scales on top of head and occiput; scales of occiput intermixed with small, rounded, tubercles; superciliaries elongate, largest dorsally; mental triangular, bordered laterally by first infralabials and posteriorly by large left and right trapezoidal postmentals contacting medially for ~ 65% of their length posterior to mental; one row of enlarged, sublabials extending posteriorly to fifth infralabials (R, L); gular and throat scales small, granular, grading posteriorly into slightly larger, flatter, smooth, imbricate, pectoral and ventral scales.

Body relatively long (AG/SVL 0.46) with well-defined ventrolateral folds; dorsal scales small, granular, interspersed with moderately sized, smooth, rounded, semi-regularly arranged tubercles extending from occiput to slightly beyond base of tail; ~ 25 longitudinal rows of tubercles at midbody; ~ 33 paravertebral tubercles; 33 flat, imbricate, ventral scales much larger than dorsal scales; eight enlarged precloacal scales, six bearing pores; no deep precloacal groove or depression; and two rows of large post-precloacal scales on midline.

Forelimbs moderate in length and stature (FL/SVL 0.16); granular scales of forelimbs slightly larger than those on body, small rounded tubercles on dorsal surface of forearms; palmar scales flat, juxtaposed; digits well-developed, inflected at basal interphalangeal joints, slightly narrower distal to inflections; subdigital lamellae transversely expanded, those proximal to joint inflections much wider than nearly unmodified lamellae distal to inflections; claws well-developed, sheathed by a dorsal and ventral scale; hind limbs robust, wider and longer than forelimbs (TBL/SVL 0.20), covered dorsally by granular scales interspersed with moderately sized tubercles, larger and flat scales anteriorly; ventral scales of thighs flat, imbricate, slightly larger than dorsals; subtibial scales small, flat, imbricate; one row of 10(R)11(L) enlarged femoral scales terminating distally before knee, continuous with enlarged precloacal scales; proximal femorals nearly same size as distal femorals, all femorals forming an abrupt union with smaller, granular, ventral scales of posteroventral scales of thigh; femoral pores 4(R) 5(L) restricted to distalmost femorals; plantar scales flat, juxtaposed; digits well-developed, inflected at basal interphalangeal joints; claws well-developed, sheathed by a dorsal and ventral scale at base; seven (R, L) wide subdigital lamellae on fourth toe proximal to joint inflection, 12 (R, L) narrower lamellae distal to joint inflection, 19 total subdigital lamellae.

Tail regenerated, long (TL/SVL 1.14), thin, 78.1 mm in length, 6.9 mm wide at base, tapering to a point; dorsal caudal scales small, generally square, juxtaposed; median row of subcaudals significantly larger than dorsal caudals, transversely expanded, not extending dorsally onto lateral side of tail; body tubercles extending slightly beyond base of tail; faint hemipenal swellings at base of tail, two large postcloacal tubercles on both sides; and postcloacal scales flat, imbricate.

##### Coloration prior to preservation.

(Figs 4, 5) Ground color of top of head, limbs, and dorsum straw to pale brown; top of head bearing poorly defined, irregularly shaped, dark brown markings; dark brown, nuchal loop bearing two posterior projections extend between postorbital regions; well-defined, rectangular dark brown band on nape; six dark brown, immaculate, weakly jagged, dorsal body bands terminating above the ventrolateral folds extending from shoulders to groin, same width as straw-colored interspaces, not edged with white or bright-colored tubercles; one darkly colored sacral band; dorsal interspaces faintly mottled, each bearing a brown “fuzzy-edged” longitudinal vertebral marking; forelimbs faintly mottled; hind limbs more darkly mottled, accentuating light-colored tubercles; one post-sacral and five wide, dark brown caudal bands slightly wider whitish caudal bands markings; whitish caudal bands do not encircle tail, bear subcircular dark brown markings; iris reddish gold with thin black reticulations; venter beige with faint, dark shadowing on lateral edges of belly and limbs; and subcaudal region dark-brown, weakly mottled with pale-colored markings.

##### Etymology.

The species name *phamiensis* is in reference to the type locality at Pha Mi Village, Wiang Phang Kham Subdistrict, Mae Sai District, Chiang Rai Province, Thailand (Fig. 1).

##### Distribution.

The type series of *Cyrtodactylusphamiensis* sp. nov. is known only from the type locality at Pha Mi Village, Wiang Phang Kham Subdistrict, Mae Sai District, Chiang Rai Province, Thailand (Fig. 1). On 30 March 2023, a bat researcher from the University of Hong Kong, Ada Chornelia, informed us of this species’ potential presence in a two adjacent karst caves at monastery 5 km north of the type locality in Tham Pha Chom along the same line of large karst formations. Examination of a photograph (Fig. 6) from this locality, tentatively confirms this observation. Furthermore, on 17 December 2022 three *Cyrtodactylusphamiensis* sp. nov. were observed, one of which was photographed, only 50 meters west-southwest of the type locality immediately east of Pha Mi Village by H. Bringsøe (Fig. 7) from the same karstic formations. It is likely that *Cyrtodactylusphamiensis* sp. nov. ranges throughout the karstic landscapes of this region.

##### Variation.

(Table 4) The paratypes closely approach the holotype in general coloration and pattern (Fig. 8). The most notable variation pertains to the shape of the nuchal loop, nape band, the caudal pattern and morphology. In ZMKU R 01073–74, ZMKU R 01081, ZMKU R 01083–84 and ZMKU R 01087, the nuchal loop is medially bifurcated. That of ZMKU R 01075 is irregular and ill-defined. The holotype, ZMKU R 01086, is the only specimen with a complete rectangular nape band. Nape bands of the paratypes are either tripartite or nearly so. ZMKU R 01074, ZMKU R 01084 and ZMKU R 01087 have complete tails. Tails of ZMKU R 01075 and ZMKU R 01078 are three-quarters to one-half regenerated, respectively. ZMKU R 01081 and ZMKU R 01085 lack the majority of their tails. The body bands of ZMKU R 01074–75, ZMKU R 01085 and ZMKU R 01087 have lightened centers. Morphometric, meristic, and categorical data of the type series and referred specimens are listed in Suppl. material 3.

##### Comparisons.

*Cyrtodactylusphamiensis* sp. nov. is embedded in clade 2 and is the sister species to a clade composed of three lineages, *C.doisuthep*, *C.erythrops* and *C.* sp. 6. *Cyrtodactylusphamiensis* sp. nov. differs from those three lineages by mean uncorrected pairwise sequence divergence of 13.5–14.5% and the remaining species in the *chauquangensis* group by 13.7–17.3% (Suppl. material 2). It differs from *C.doisuthep* by having maximum SVL 74.4 mm (vs 90.5 mm); 9–14 total number of femoral pores in males (vs absent); and lacking light-colored reticulum on the top of the head (vs present; Kunya et al. 2014: fig. 1). *Cyrtodactylusphamiensis* sp. nov. differs from *C.erythrops* by having 29–37 longitudinal rows of ventrals (vs 28 rows); 9–14 total number of femoral pores in males (vs 19 pores); 4–6 precloacal pores in males (vs 9 pores); and hatchlings with yellow-colored heads (vs tan colored; Bauer et al. 2009: fig. 5). Additional comparisons (meristics, morphometrics, and subcaudal scale morphology) between *Cyrtodactylusphamiensis* sp. nov. and the remaining species in the *chauquangensis* group are presented in Table 4.

##### Natural history.

**Figure 9.**
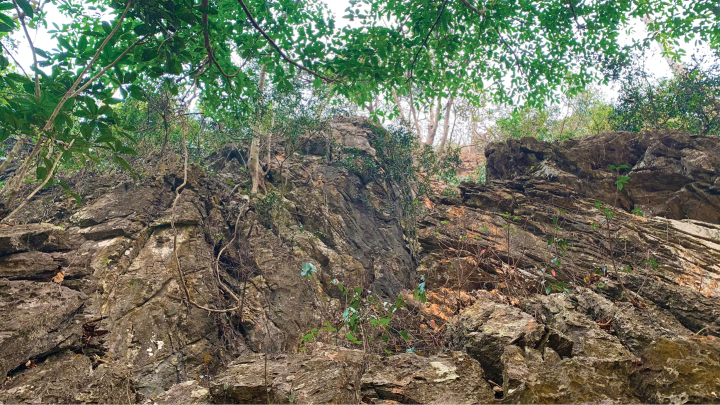
Karst habitat of the type locality from Pha Mi Village, Wiang Phang Kham Subdistrict, Mae Sai District, Chiang Rai Province, Thailand. Photograph by Evan S.H. Quah.

(Fig. 9) All specimens were collected during the evening between 19:30 and 20:50 hours on karst formations, at the entrance of a karst cave, within the cave, or on karst vegetation outside the cave at varying distances from the cave entrance. One specimen was observed during the day in a crack ~ 5 m above the cave floor ~ 20 m in from the entrance. Juveniles (SVL < 40 mm) were found outside the cave less than 1 m above ground level on karst boulders or on the base of small trees. Most were found farther away (~ 20–40 m) from the cave entrance than adults. On 26 March, four or five juveniles (not collected) were also observed far from the cave entrance on karst boulders and on the base of trees. That same night, other juveniles were observed near ground level on small karst outcroppings along a shallow ravine ~ 0.3 m southeast of the type locality. We have noted similar behavior in juveniles of *Cyrtodactylusaunglini* Grismer, Wood, Thura, Win, Grismer, Trueblood & Quah, 2018, *C.bayinnyiensis* Grismer, Wood, Thura, Quah, Murdoch, Grismer, Herr, Lin & Kyaw, 2018, *C.chrysopylos* Bauer, 2003, and *C.shwetaungorum* Grismer, Wood, Thura, Zin, Quah, Murdoch, Grismer, Lin, Kyaw & Lwin, 2017, of unrelated species groups in Myanmar (Grismer et al. 2018a, 2018b, 2018c). All hatchlings of these were found on the ground far from the adults on karst formations. We suspect this may be a way to avoid predation by adults as well as a means to disperse to other karst habitats. The fact that several juveniles of *Cyrtodactylusphamiensis* sp. nov. and no gravid females were observed indicates the reproductive season must have terminated prior to March. The three individuals of *Cyrtodactylusphamiensis* sp. nov. which were found 50 meters from the type locality on 17 December 2022 were adults and were observed on the karst walls outside caves at night between 21:30 and 22:30 hours (Fig. 7).

Other species of herpetofauna observed in the vicinity during this period were two species of frogs, *Sylvirananigrovittata* (Blyth, 1856) and *Polypedatesmegacephalus* Hallowel, 1861; four other gecko species, *Gehyramutilata* (Wiegmann, 1834), *Gekkogecko* (Linnaeua, 1758), *Hemidactylusgarnotii* Duméril & Bibron, 1836, and *Hemidactylusplatyurus* (Schneider, 1797); and a pitviper *Trimeresurusmacrops* Kramer, 1977. We postulate that the high number of adult *Cyrtodactylusphamiensis* sp. nov. that had missing or regenerated tails as well as their skittish nature and that they did not stray far from their shelters could have been due to predation pressures from the large *G.gecko* that were also found on the karst walls and the pitvipers that were observed coiled in ambush position on vegetation beside the karst.

## ﻿Discussion

The computation of nine phylogenies from three model-based analyses using three different partition schemes did not resolve all of the unequivocal nodes variably present in the most recent analyses (Liu and Rao 2021a, 2021b, 2022; Liu et al. 2021; Chomdej et al. 2022; Tran et al. 2024). Direct comparison to the previously published trees is difficult because one used CO1 instead of ND2 (Liu and Rao 2021b) and the others had incomplete species coverage of the group. Our analysis also lacked *Cyrtodactyluscaixitaoi* Liu, Rao, Hou, Wang & Ananjeva, 2023, *C.hekouensis* Zhang, Liu, Bernstein, Wang & Yuan, 2021, and *C.martini* Ngo, 2011 (Ngo 2011; Zhang et al. 2021; Liu et al. 2023). Nonetheless, running multiple analyses underscores the necessity to evaluate the performance of more than just one or two models employing a single partition scheme. Had we only run codon-partitioned ML and BI analyses – which is generally standard for most integrative taxonomic studies in herpetology although many drop the BI analysis – we would not have recovered the best resolved tree. Had we not employed the BEAST analysis, the ML analysis would have generated the best phylogeny but with five fewer strongly supported nodes. However, BEAST analyses can inflate nodal support values. With *a priori* knowledge that many of the internal nodes of the clade being tested are not well-supported, running varying partition schemes across different models offers the best chance of recovering the best tree possible given the data. Additionally, it should be noted that if the marker used is highly informative, then all phylogenetic iterations should recover the same well supported phylogeny.

The topology of the tree generated herein is similar to that of Liu and Rao (2022) based on their codon partitioned ML and BI although they recovered five deep nodes with no support as opposed three unsupported nodes herein. The topologies of these trees were inconsistent with that of Chomdej et al. (2022) which used one substitution model for both genes (ND2 and tRNA) in their ML and BI analyses and did not recover the *chauquangensis* group as monophyletic. The differences in the latter case may be due to the choice of outgroups used to root the trees. For the most part, the unresolved nodes in all the trees occur at the ends of deep short internodes, indicating speciation in this part of the trees was rapid as opposed to other parts (Fig. 2). Until other markers are used to resolve these equivocal nodes, the current phylogeny here falls outside the legitimate purview of any comparative phylogenetic methods for character evolution, biogeography, evolution of habitat preference, etc., – all of which could potentially affect plans for conservation management.

The discovery of new species of *Cyrtodactylus* in karstic caves, towers, cones, or hills in Southeast Asia and Indochina has become more of an expectation than a surprise and vast areas of karstic landscapes across these regions remain unexplored. These landscapes are proving to have a far greater number of species across the taxonomic board than previously expected. This is especially true for *Cyrtodactylus* where karst landscapes have been shown to be foci of speciation (Grismer et al. 2021) as opposed to only being refugial “arks”. The growing research in karstic landscapes will continue to underscore their unrealized biodiversity, further emphasizing the need for their conservation, something they woefully lack across all of Asia.

## Supplementary Material

XML Treatment for Cyrtodactylus phamiensis

## ﻿Additional information

### Author ORCIDs

L. Lee Grismer https://orcid.org/0000-0001-8422-3698

Anchalee Aowphol https://orcid.org/0000-0001-9504-4601

Jesse L. Grismer https://orcid.org/0000-0002-2542-5430

Akrachai Aksornneam https://orcid.org/0000-0003-4780-376X

Evan S. H. Quah https://orcid.org/0000-0002-5357-1953

Matthew L. Murdoch https://orcid.org/0000-0001-5914-6408

Attapol Rujirawan https://orcid.org/0000-0001-9179-6910

### Data availability

All of the data that support the findings of this study are available in the main text or Supplementary Information.

## Supplementary materials

Supplementary material 1 GenBank accession numbers for the mitochondrial NADH dehydrogenase subunit 2 (ND2) gene and catalog number of voucher specimens used in this analysis Data type pdf https://binary.pensoft.net/file/1059378This dataset is made available under the Open Database License (http://opendatacommons.org/licenses/odbl/1.0/). The Open Database License (ODbL) is a license agreement intended to allow users to freely share, modify, and use this Dataset while maintaining this same freedom for others, provided that the original source and author(s) are credited.L. Lee Grismer, Anchalee Aowphol, Jesse L. Grismer, Akrachai Aksornneam, Evan S. H. Quah, Mathew L. Murdoch, Jeren J. Gregory, Eddie Nguyen, Amanda Kaatz, Henrik Bringsøe, Attapol Rujirawan

Supplementary material 2 Mean uncorrected pairwise genetic distance (%) between species of the *Cyrtodactyluschauquangensis* group based on the mitochondrial NADH dehydrogenase subunit 2 (ND2) gene Data type pdf https://binary.pensoft.net/file/1059379This dataset is made available under the Open Database License (http://opendatacommons.org/licenses/odbl/1.0/). The Open Database License (ODbL) is a license agreement intended to allow users to freely share, modify, and use this Dataset while maintaining this same freedom for others, provided that the original source and author(s) are credited.L. Lee Grismer, Anchalee Aowphol, Jesse L. Grismer, Akrachai Aksornneam, Evan S. H. Quah, Mathew L. Murdoch, Jeren J. Gregory, Eddie Nguyen, Amanda Kaatz, Henrik Bringsøe, Attapol Rujirawan

Supplementary material 3 Morphological and color pattern data for the type series and hatchlings of *Cyrtodactylusphamiensis* sp. nov. Data type pdf Explanation note Key: / = data unavailable or inapplicable; m = male; f = female; r = regenerated; b = broken; y = yes; *n* = no. https://binary.pensoft.net/file/1059380This dataset is made available under the Open Database License (http://opendatacommons.org/licenses/odbl/1.0/). The Open Database License (ODbL) is a license agreement intended to allow users to freely share, modify, and use this Dataset while maintaining this same freedom for others, provided that the original source and author(s) are credited.L. Lee Grismer, Anchalee Aowphol, Jesse L. Grismer, Akrachai Aksornneam, Evan S. H. Quah, Mathew L. Murdoch, Jeren J. Gregory, Eddie Nguyen, Amanda Kaatz, Henrik Bringsøe, Attapol Rujirawan
