# Supplementary material for: ﻿A new species of the Cyrtodactyluschauquangensis group (Squamata, Gekkonidae) from the borderlands of extreme northern Thailand
Source: Zookeys. 2024 May 30;1203:211–38. doi: 10.3897/zookeys.1203.122758 (PMC11161685; doi:10.3897/zookeys.1203.122758)
Supplement: Supplementary material 1 — GenBank accession numbers for the mitochondrial NADH dehydrogenase subunit 2 (ND2) gene and catalog number of voucher specimens used in this analysis [file zookeys-1203-211_article-122758__-s001.pdf]

Supplementary material 1. GenBank accession numbers for the mitochondrial NADH dehydrogenase subunit 2 (ND2) gene and catalog number of voucher specimens used in this analysis.

Species	Locality	Catalog number	GenBank accesion number	Reference
Cyrtodactylus auribalteatus	Thailand, Phitsanulok Province, Noen Maprang District	AUP-01745	MZ439914	Chomdej et al. (2022)
Cyrtodactylus auribalteatus	Thailand, Phitsanulok Province, Noen Maprang District	AUP-01746	MZ439915	Chomdej et al. (2022)
Cyrtodactylus bichnganae	Vietnam, Son La Province, Son La Urban	UNS 0473	MF169953	Brennan et al. (2017)
Cyrtodactylus bobrovi	Vietnam, Hoa Binh Province, Ngoc Son–Ngo Luong Nature Reserve	IEBR A.2015.29	MT953471	Grismer et al. (2020)
Cyrtodactylus chauquangensis	Vietnam, Nghe An Province, Quy Hop District	NA 2016.1	MT953475	Grismer et al. (2020)
Cyrtodactylus cucphuongensis	Vietnam, Ninh Binh Province, Cuc Phuong NP	CP 17.02	MT953477	Grismer et al. (2020)
Cyrtodactylus dammathetensis	Myanmar, Mon State, Mawlamyine District	LSUHC:12863	MF872277	Grismer et al. (2018)
Cyrtodactylus doisuthep	Thailand, Chiang Mai Province, Doi Suthep	AUP-00777	MT497801	Chomdej et al. (2021)
Cyrtodactylus doisuthep	Thailand, Chiang Mai Province, Doi Suthep	AUP-00774	MT550626	Chomdej et al. (2020)
Cyrtodactylus dumnuii	Thailand, Chiang Mai Province, Chiang Dao District	AUP-00769	MT497802	Chomdej et al. (2021)
Cyrtodactylus dumnuii	Thailand, Chiang Mai Province, Chiang Dao District	AUP-00770	MT497803	Chomdej et al. (2021)
Cyrtodactylus dumnuii	Thailand, Chiang Mai Province, Chiang Dao District	AUP 00768	MW713972	Grismer et al. (2021)
Cyrtodactylus erythrops	Thailand, Mae Hong Son province, Pang Mapha District	AUP-00771	MT497806	Chomdej et al. (2021)
Cyrtodactylus erythrops	Thailand, Mae Hong Son province, Pang Mapha District	AUP 00772	MW713958	Grismer et al. (2021)
Cyrtodactylus gulinqingensis	China, Yunnan Province, Maguan County, Gulinqing Nature Reserve	KIZ 061813	MZ782150	Liu et al. (2021)
Cyrtodactylus gulinqingensis	China, Yunnan Province, Maguan County, Gulinqing Nature Reserve	KIZ 061814	MZ782151	Liu et al. (2021)
Cyrtodactylus gulinqingensis	China, Yunnan Province, Maguan County, Gulinqing Nature Reserve	KIZ 061816	MZ782152	Liu et al. (2021)
Cyrtodactylus gulinqingensis	China, Yunnan Province, Maguan County, Gulinqing Nature Reserve	KIZ 061817	MZ782153	Liu et al. (2021)
Cyrtodactylus houaphanensis	Laos, Luang Houaphan Province	IEBR A.2013.109	MW792067	Grismer et al. (2021)
Cyrtodactylus huongsonensis	Vietnam, Ha Noi, My Duc District, Huong Son	IEBR A.2011.3A	MT953481	Grismer et al. (2020)
Cyrtodactylus kunyai	Thailand, Loei Province, Nong Hin District	AUP-01747	MZ439916	Chomdej et al. (2022)
Cyrtodactylus kunyai	Thailand, Loei Province, Nong Hin District	AUP-01748	MZ439917	Chomdej et al. (2022)
Cyrtodactylus luci	Vietnam, Lao Cai Province, Bac Ha District	IEBR R.5240	PP253960	Tran et al. (2024)
Cyrtodactylus luci	Vietnam, Lao Cai Province, Bac Ha District	IEBR R.5241	PP253959	Tran et al. (2024)
Cyrtodactylus menglianensis	China, Yunnan Province, Puer City, Menglian County	KIZ 20210713	OM296042	Liu and Rao (2022)
Cyrtodactylus menglianensis	China, Yunnan Province, Puer City, Menglian County	KIZ 20210716	OM296044	Liu and Rao (2022)
Cyrtodactylus ngoiensis	Laos, Luang Prabang Province, Ngoi District	IEBR A.2013.110	MW792066	Grismer et al. (2021)
Cyrtodactylus otai	Vietnam, Son La Province, Van Ho District, Na Bai Village	TBU 2017.2	MT953486	Grismer et al. (2020)
Cyrtodactylus phamiensis sp. nov.	Thailand, Chiang Rai Province, Mae Sai District, Pha Mi Village	ZMKU R 01074	PP430583	This study
Cyrtodactylus phamiensis sp. nov.	Thailand, Chiang Rai Province, Mae Sai District, Pha Mi Village	ZMKU R 01075	PP430586	This study
Cyrtodactylus phamiensis sp. nov.	Thailand, Chiang Rai Province, Mae Sai District, Pha Mi Village	ZMKU R 01076	PP430584	This study
Cyrtodactylus phamiensis sp. nov.	Thailand, Chiang Rai Province, Mae Sai District, Pha Mi Village	ZMKU R 01079	PP430588	This study
Cyrtodactylus phamiensis sp. nov.	Thailand, Chiang Rai Province, Mae Sai District, Pha Mi Village	ZMKU R 01080	PP430587	This study
Cyrtodactylus phamiensis sp. nov.	Thailand, Chiang Rai Province, Mae Sai District, Pha Mi Village	ZMKU R 01081	PP430589	This study
Cyrtodactylus phamiensis sp. nov.	Thailand, Chiang Rai Province, Mae Sai District, Pha Mi Village	ZMKU R 01082	PP430591	This study
Cyrtodactylus phamiensis sp. nov.	Thailand, Chiang Rai Province, Mae Sai District, Pha Mi Village	ZMKU R 01084	PP430590	This study
Cyrtodactylus phamiensis sp. nov.	Thailand, Chiang Rai Province, Mae Sai District, Pha Mi Village	ZMKU R 01085	PP430582	This study
Cyrtodactylus phamiensis sp. nov.	Thailand, Chiang Rai Province, Mae Sai District, Pha Mi Village	ZMKU R 01086	PP430585	This study
Cyrtodactylus phukhaensis	Thailand, Nan Province, Pua District, Doi Phu Kha	KIZ 042649/AUP-01823	MZ439912	Chomdej et al. (2022)
Cyrtodactylus phukhaensis	Thailand, Nan Province, Pua District, Doi Phu Kha	KIZ 042652/AUP-01824	MZ439913	Chomdej et al. (2022)
Cyrtodactylus puhuensis	Vietnam, Thanh Hoa Province	ND 01.15	MT953489	Grismer et al. (2020)
Cyrtodactylus sinyineensis	Myanmar, Kayin State, Hpa-an District	LSUHC:12836	MF872355	Grismer at al. (2018)
Cyrtodactylus solaensis	Vietnam, Son La Province, Phu Yen District	IEBR A.2017.1	MT953492	Grismer et al. (2020)
Cyrtodactylus soni	Vietnam, Ninh Binh Province, Gia Vien District	IEBR R.2016.4	MT953491	Grismer et al. (2020)
Cyrtodactylus sp. 6	Thailand, Mae Hong Son Province, Mae Hong Son Mueang District	AUP-01576	MT468908	Chomdej et al. (2021)

Species	Locality	Catalog number	GenBank accesion number	Reference
Cyrtodactylus spelaeus	Laos, Vientiane Province, Kasi	HLM 0315	MW713962	Grismer et al. (2021)
Cyrtodactylus taybacensis	Vietnam, Son La Province, Quyun Nhai District, Ca Nang Village	IEBR 4379	MT953495	Grismer et al. (2020)
Cyrtodactylus vilaphongi	Laos, Luang Prabang Province, Luang Prabang District	IEBR A.2013.103	MT953497	Grismer et al. (2020)
Cyrtodactylus wayakonei	Laos, Luang Nam Tha Province, Vieng Phoukha District	ZFMK 91016	MT953498	Grismer et al. (2020)
Cyrtodactylus zhenkangensis	China, Yunnan Province, Lincang City, Zhenkang County	KIZL2020047	MW792062	Grismer et al. (2021)

Reference

Brennan IG, Bauer AM, Van Tri N, Wang YY, Wang WZ, Zhang YP, Murphy RW (2017) Barcoding utility in a mega-diverse, cross-continental genus: keeping pace with *Cyrtodactylus* geckos. Scientific Reports 7(1):5592. https://doi.org/10.1038/s41598-017-05261-9.

Chomdej S, Suwannapoom C, Pawangkhanant P, Pradit W, Nazarov RA, Grismer LL, Poyarkov NA (2020) A new species *Cyrtodactytlus* Gray (Squamata: Gekkonidae) from western Thailand and the phylogenetic placement of *C. inthanon* and *C. doisuthep.* Zootaxa 4838 (2): 179–209. https://doi.org/10.11646/zootaxa.4838.2.2

Chomdej S, Pradit W, Suwannapoom C, Pawangkhanant P, Nganvongpanit K, Poyarkov NA, Che J, Gao YC, Gong SP (2021) Phylogenetic analyses of distantly related clades of bent-toed geckos (genus *Cyrtodactylus*) reveal an unprecedented amount of cryptic diversity in northern and western Thailand. Scientific Reports 11(1): e2328. https://doi.org/10.1038/s41598-020-70640-8

Chomdej S, Pradit W, Pawangkhanant P, Kuensaen C, Phupanbai A, Naiduangchan M, Piboon P, Nganvongpanit K, Yuan Z, Zhang Y, Che J, Cucharitakul P, Suwannapoom C (2022) A New Cytrodactylus species (Reptilia: Gekkonidae) from Nan Province, Northern Thailand. Asian Herpetological Research 13: 96–108. https://doi.org/10.16373/j.cnki.ahr.210055

Grismer LL, Wood Jr PL, Thura MK, Zin T, Quah ESH, Murdoch ML, Grismer MS, Lin A, Kyaw H, Lwin N (2018) Twelve new species of *Cyrtodactylus* Gray (Squamata: Gekkonidae) from isolated limestone habitats in east-central and southern Myanmar demonstrate high localized diversity and unprecedented microendemism. Zoological Journal of the Linnean Society 182: 862–959. https://doi.org/10.1093/zoolinnean/zlx057

Grismer LL, Wood PL, Le MD, Quah ESH, Grismer JL (2020) Evolution of habitat preference in 243 species of Bent-toed geckos (Genus *Cyrtodactylus* Gray, 1827) with a discussion of karst habitat conservation. Ecology and Evolution 10(24): 13717-13730. https://doi.org/10.1002/ece3.6961

Grismer LL, Wood Jr PL, Poyarkov NA, Le MD, Kraus F, Agarwal I, Oliver PM, Nguyen SN, Nguyen TQ, Karunarathna S, Welton LJ, Stuart BL, Luu VQ, Bauer AM, O'Connell KA, Quah ESH, Chan KO, Ziegler T, Ngo H, Nazarov RA, Aowphol A, Chomdej S, Suwannapoom C, Siler CD, Anuar S, Tri NV, Grismer JL (2021) Phylogenetic partitioning of the third-largest vertebrate genus in the world, *Cyrtodactylus* Gray, 1827 (Reptilia; Squamata; Gekkonidae) and its relevance to taxonomy and conservation. Vertebrate Zoology 71: 101–154. https://doi.org/10.3897/vertebrate-zoology.71.e59307

Liu S, Rao D (2022) A new species of *Cyrtodactylus* Gray, 1827 (Squamata, Gekkonidae) from southwestern Yunnan, China. ZooKeys 1084: 83–100. https://doi.org/10.3897/zookeys.1084.72868

Liu S, Qisheng L, Mian H, Orlov N, Ananjeva NB (2021) A New Species of *Cyrtodactylus* Gray, 1827 (Squamata, Gekkonidae) from Southern Yunnan, China. Russian Journal of Herpetology 28: 185–196. https://doi.org/10.30906/1026-2296-2021-28-4-185-196

Tran TT, Do QH, Pham CT, Phan TQ, Ngo HT, Le MD, Ziegler T, Nguyen TQ (2024) A new species of the *Cyrtodactylus chauquangensis* species group (Squamata, Gekkonidae) from Lao Cai Province, Vietnam. ZooKeys 1192:83–102. https://doi.org/10.3897/zookeys.1192.117135
