# Supplementary material for: ﻿A new species of the Cyrtodactyluschauquangensis group (Squamata, Gekkonidae) from the borderlands of extreme northern Thailand
Source: Zookeys. 2024 May 30;1203:211–38. doi: 10.3897/zookeys.1203.122758 (PMC11161685; doi:10.3897/zookeys.1203.122758)
Supplement: Supplementary material 2 — Mean uncorrected pairwise genetic distance (%) between species of the Cyrtodactyluschauquangensis group based on the mitochondrial NADH dehydrogenase subunit 2 (ND2) gene [file zookeys-1203-211_article-122758__-s002.pdf]

Supplemetary material 2. Mean uncorrected pairwise genetic distance (%) between species of the *Cyrtodactylus chauquangensis* group based on the mitochondrial NADH dehydrogenase subunit 2 (ND2) gene.

**Species**	***Cyrtodactylus auribalteatus***	***Cyrtodactylus bichnganae***	***Cyrtodactylus bobrovi***	***Cyrtodactylus chauquangensis***	***Cyrtodactylus cucphuongensis***	***Cyrtodactylus doisuthep***	***Cyrtodactylus dumnuii***	***Cyrtodactylus erythrops***	***Cyrtodactylus gulinqingensis***	***Cyrtodactylus houaphanensis***	***Cyrtodactylus huongsonensis***	***Cyrtodactylus menglianensis***	***Cyrtodactylus ngoiensis***	***Cyrtodactylus otai***	***Cyrtodactylus phamiensis* sp. nov.**	***Cyrtodactylus phukhaensis***	***Cyrtodactylus puhuensis***	***Cyrtodactylus soni***	***Cyrtodactylus sonlaensis***	***Cyrtodactylus* sp. 6**	***Cyrtodactylus spelaeus***	***Cyrtodactylus taybacensis***	***Cyrtodactylus vilaphongi***	***Cyrtodactylus wayakonei***	***Cyrtodactylus zhenkangensis***
Cyrtodactylus auribalteatus	–																								
Cyrtodactylus bichnganae	17.79	–																							
Cyrtodactylus bobrovi	14.95	19.92	–																						
Cyrtodactylus chauquangensis	14.33	18.11	8.59	–																					
Cyrtodactylus cucphuongensis	15.76	19.92	7.87	8.42	–																				
Cyrtodactylus doisuthep	14.27	16.69	15.72	14.26	15.61	–																			
Cyrtodactylus dumnuii	12.82	16.68	13.57	12.11	14.00	13.92	–																		
Cyrtodactylus erythrops	14.67	17.21	14.73	13.50	14.71	10.89	13.34	–																	
Cyrtodactylus gulinqingensis	13.87	17.85	13.37	13.73	13.64	13.44	12.33	13.46	–																
Cyrtodactylus houaphanensis	15.45	19.66	6.47	9.00	7.53	15.38	14.07	14.94	13.71	–															
Cyrtodactylus huongsonensis	14.42	17.72	14.25	12.43	14.29	14.81	13.77	14.28	12.13	14.74	–														
Cyrtodactylus menglianensis	12.42	18.76	14.96	12.68	14.98	15.30	11.09	13.98	13.95	14.75	14.42	–													
Cyrtodactylus ngoiensis	13.64	18.37	11.14	10.42	10.70	14.90	11.67	14.23	12.81	11.33	13.07	12.99	–												
Cyrtodactylus otai	14.18	19.40	3.58	9.10	8.44	16.26	15.22	16.42	15.23	6.81	14.73	15.25	12.24	–											
Cyrtodactylus phamiensis sp.nov.	14.94	15.79	15.12	14.18	15.57	13.48	13.72	14.49	14.83	14.98	14.01	15.10	14.55	16.10	–										
Cyrtodactylus phukhaensis	12.52	17.55	14.52	11.84	14.23	14.86	11.55	13.97	13.54	14.44	13.78	7.02	11.11	15.33	14.24	–									
Cyrtodactylus puhuensis	14.28	19.40	5.67	8.00	7.13	14.63	12.65	14.18	13.20	2.83	13.84	14.13	10.46	6.17	14.49	13.67	–								
Cyrtodactylus soni	13.70	18.38	14.37	13.51	14.19	13.98	12.83	14.09	12.65	15.33	6.82	13.60	14.13	14.91	13.98	13.93	14.36	–							
Cyrtodactylus sonlaensis	16.18	19.61	17.47	16.79	18.15	16.26	16.57	17.28	14.33	17.95	14.96	17.18	16.22	17.66	17.34	16.03	17.95	15.19	–						
Cyrtodactylus sp. 6	14.47	18.24	14.38	14.58	14.34	11.59	13.83	6.87	13.47	15.03	14.58	14.72	13.65	15.62	13.83	14.14	14.63	14.11	17.76	–					
Cyrtodactylus spelaeus	15.12	18.37	10.15	9.17	10.15	15.38	13.13	14.87	13.41	10.66	14.24	14.57	11.31	11.29	15.22	13.61	9.38	14.29	17.47	15.27	–				
Cyrtodactylus taybacensis	17.31	9.72	16.63	15.51	16.90	15.48	14.62	16.07	15.72	16.98	15.77	16.67	16.41	18.33	16.19	16.88	16.29	15.31	18.94	15.85	16.21	–			
Cyrtodactylus vilaphongi	17.31	17.98	8.15	7.25	8.16	14.20	12.95	13.91	13.22	8.18	14.16	14.04	9.49	9.05	14.29	13.71	7.05	13.37	16.89	13.64	9.76	16.10	–		
Cyrtodactylus wayakonei	13.26	18.24	15.49	13.09	15.45	16.29	12.23	15.59	14.99	14.74	15.06	7.18	12.17	15.42	14.77	4.91	14.22	14.10	16.41	15.59	15.28	17.47	13.69	–	
Cyrtodactylus zhenkangensis	12.70	19.02	14.15	13.08	13.74	15.20	11.57	14.10	12.55	13.91	13.32	10.77	13.20	15.52	14.90	10.15	13.23	13.87	17.28	14.32	14.07	15.40	13.62	11.93	–
