# Supplementary material for: ﻿A new species of the Cyrtodactyluschauquangensis group (Squamata, Gekkonidae) from the borderlands of extreme northern Thailand
Source: Zookeys. 2024 May 30;1203:211–38. doi: 10.3897/zookeys.1203.122758 (PMC11161685; doi:10.3897/zookeys.1203.122758)
Supplement: Supplementary material 3 — Morphological and color pattern data for the type series and hatchlings of Cyrtodactylusphamiensis sp. nov. [file zookeys-1203-211_article-122758__-s003.pdf]

**Supplementary material 3.** Morphological and color pattern data for the type series and hatchlings of *Cyrtodactylus phamiensis* sp. nov. **Key:** / = data unavailable or inapplicable; m = male; f = female; r = regenerated; b = broken; y = yes; n = no.

| | **ZMKU R 01086** | **ZMKU R 01073** | **ZMKU R 01074** | **ZMKU R 01075** | **ZMKU R 01078** | **ZMKU R 01081** | **ZMKU R 01084** | **ZMKU R 01085** | **ZMKU R 01087** | **ZMKU R 01076** | **ZMKU R 01077** | **ZMKU R 01079** | **ZMKU R 01080** | **ZMKU R 01082** | **ZMKU R 01083** |
|---|---|---|---|---|---|---|---|---|---|---|---|---|---|---|---|
| **Sex** | m | f | f | f | f | m | f | m | m | hatchling | hatchling | hatchling | hatchling | hatchling | hatchling |
| **Type** | holotype | paratype | paratype | paratype | paratype | paratype | paratype | paratype | paratype | | | | | | |
| **Meristics** | | | | | | | | | | | | | | | |
| Supralabials (SL) | 9 | 9 | 10 | 9 | 11 | 8 | 9 | 8 | 9 | 11 | 9 | 10 | 10 | 10 | 12 |
| Infralabials (IL) | 11 | 10 | 10 | 11 | 10 | 9 | 9 | 10 | 10 | 9 | 9 | 10 | 9 | 10 | 10 |
| Paravertebral tubercles (PVT) | 33 | 31 | 32 | 35 | 37 | 31 | 40 | 30 | 32 | 33 | 43 | 34 | 31 | 33 | 39 |
| Longitudinal rows of body tubercles (LRT) | 25 | 23 | 20 | 21 | 19 | 23 | 20 | 21 | 19 | 23 | 24 | 23 | 23 | 21 | 22 |
| Ventral scales (VS) | 33 | 31 | 30 | 35 | 29 | 33 | 34 | 30 | 36 | 37 | 32 | / | 36 | 31 | 32 |
| Expanded subdigital lamellae on 4th toe (E4TL) | 7 | 8 | 6 | 8 | 9 | 7 | 7 | 8 | 8 | 8 | 7 | 7 | 6 | 9 | 8 |
| Unmodified subdigital lamellae on 4th toe (U4TL) | 12 | 13 | 13 | 12 | 13 | 13 | 14 | 12 | 13 | 12 | 12 | 13 | 13 | 12 | 12 |
| Total subdigital lamellae on 4th toe (T4TL) | 19 | 21 | 19 | 20 | 22 | 20 | 21 | 20 | 21 | 20 | 19 | 20 | 19 | 21 | 20 |
| Enlarged precloacal scales (PS) | 8 | 9 | 8 | 7 | 7 | 8 | 9 | 6 | 8 | 10 | 9 | 8 | 8 | 11 | 8 |
| Precloacal pores (PP) | 6 | / | / | / | / | 6 | / | 4 | 6 | / | / | / | / | / | / |
| Enlarged post-precloacal scale rows (PPS) | 2 | 3 | 2 | 2 | 3 | 3 | 3 | 2 | 3 | 2 | 2 | 2 | 2 | 3 | 2 |
| Enlarged femoral scales (right/left) | 10/11 | 13/13 | 12/10 | 12/11 | 13/14 | 12/12 | 13/13 | 13/10 | 12/12 | 13/12 | 12/13 | 13/15 | 12/13 | 13/12 | 8/11 |
| Total femoral scales (FS) | 21 | 26 | 22 | 23 | 27 | 24 | 26 | 23 | 24 | 25 | 25 | 28 | 25 | 25 | 19 |
| Femoral pores (right/left) | 4/5 | / | / | / | / | 7/7 | / | 5/5 | 6/5 | / | / | / | / | / | / |
| Total femoral pores in males (PP) | 9 | / | / | / | / | 14 | / | 10 | 11 | / | / | / | / | / | / |
| Number of body bands (BB) | 6 | 6 | 5 | 4 | 6 | 6 | 5 | 4 | 5 | 5 | 5 | 5 | 5 | 5 | 5 |
| Number of light caudal bands (LCB) | 5r | 5r | 12 | 1r | 3r | / | 11 | / | 12 | 10 | 11 | 12 | / | 13 | / |
| Number of dark caudal bands (DCB) | 5r | 6r | 11 | 1r | 2r | / | 11 | / | 10 | 9 | 11 | 12 | / | 12 | / |
| **Morphology** | | | | | | | | | | | | | | | |
| Tubercles on forelimbs | small | small | small | small | small | small | small | small | small | small | small | small | small | small | small |
| Tubercles extend beyond base of tail | y | y | y | y | y | y | y | y | y | y | y | y | y | y | y |

| | ZMKU R 01086 | ZMKU R 01073 | ZMKU R 01074 | ZMKU R 01075 | ZMKU R 01078 | ZMKU R 01081 | ZMKU R 01084 | ZMKU R 01085 | ZMKU R 01087 | ZMKU R 01076 | ZMKU R 01077 | ZMKU R 01079 | ZMKU R 01080 | ZMKU R 01082 | ZMKU R 01083 |
|---|---|---|---|---|---|---|---|---|---|---|---|---|---|---|---|
| **Sex** | m | f | f | f | f | m | f | m | m | hatchling | hatchling | hatchling | hatchling | hatchling | hatchling |
| **Type** | holotype | paratype | paratype | paratype | paratype | paratype | paratype | paratype | paratype | | | | | | |
| Femoral pores restricted to distal scales | y | / | / | / | / | y | / | y | y | / | / | / | / | / | / |
| Body tubercles low, smooth to weakly keeled | y | y | y | y | y | y | y | y | y | y | y | y | y | y | y |
| Body tubercles raised, moderately to strongly keeled | n | n | n | n | n | n | n | n | n | n | n | n | n | n | n |
| Enlarged femoral and precloacal scales continuous | y | y | y | y | y | y | y | y | y | y | y | y | y | y | y |
| Pore-bearing femoral and precloacal scales continuous | n | / | / | / | / | n | / | n | n | / | / | / | / | / | / |
| Enlarged proximal femoral scales ~1/2 size of distal femorals | n | y | y | y | n | n | y | n | n | y | y | y | n | y | n |
| Medial subcaudals 2 or 3 times wider than long | y | y | y | y | y | / | y | y | y | y | y | y | / | y | y |
| Medial subcaudals extend onto lateral surface of tail | n | n | n | n | n | / | n | n | n | n | n | n | / | n | n |
| **Color pattern** | | | | | | | | | | | | | | | |
| Nuchal loop divided medially | n | y | y | n | n | y | n | n | y | y | n | y | n | y | n |
| 2 posterior projections from nuchal loop | y | n | n | n | y | n | y | y | n | n | y | n | y | n | y |
| Nuchal loop with anterior azygous notch | n | n | n | n | n | n | n | n | n | n | n | n | n | n | n |
| Triangular marking anterior to nuchal loop | n | y | y | n | n | n | n | n | n | n | n | n | n | y | n |
| Posterior border of nuchal loop | projected | smooth | smooth | jagged | projected | projected | projected | projected | projected | projected | projected | projected | projected | projected | projected |
| Band on nape | y | n | n | n | n | y | n | n | n | n | n | y | y | n | n |
| Dorsal banding with paravertebral elements | n | n | n | n | n | n | n | n | n | n | n | n | n | n | y |
| Dorsal body bands wider than interspaces | same | y | y | y | y | same | y | n | same | same | same | same | y | same | y |
| Dorsal body bands with lightened centers | n | n | y | y | n | n | n | y | y | n | n | n | n | y | n |
| Dorsal bands edged with white tubercles | n | n | n | n | n | n | n | n | n | n | y | y | y | y | n |

| | **ZMKU R 01086** | **ZMKU R 01073** | **ZMKU R 01074** | **ZMKU R 01075** | **ZMKU R 01078** | **ZMKU R 01081** | **ZMKU R 01084** | **ZMKU R 01085** | **ZMKU R 01087** | **ZMKU R 01076** | **ZMKU R 01077** | **ZMKU R 01079** | **ZMKU R 01080** | **ZMKU R 01082** | **ZMKU R 01083** |
|---|---|---|---|---|---|---|---|---|---|---|---|---|---|---|---|
| **Sex** | m | f | f | f | f | m | f | m | m | hatchling | hatchling | hatchling | hatchling | hatchling | hatchling |
| **Type** | holotype | paratype | paratype | paratype | paratype | paratype | paratype | paratype | paratype | | | | | | |
| Shape of dorsal bands | weakly jagged | straight | jagged | jagged | jagged | jagged | straight | jagged | jagged | jagged | jagged | jagged | jagged | jagged | straight |
| Dark markings in dorsal interspaces | y | y | y | y | y | n | y | y | faint | y | y | y | y | y | y |
| Top of head diffusely mottled, blotched | y | y | y | y | y | y | y | y | y | y | y | y | y | y | y |
| Light-colored reticulum on top of head | n | n | n | n | n | n | n | n | n | n | n | n | n | n | n |
| White caudal bands with dark markings | y | y | y | y | y | / | y | / | faint | y | y | y | / | y | y |
| White caudal bands encircle tail | n | n | n | n | n | / | n | / | n | n | n | n | / | n | n |
| Dark caudal bands wider than light caudal bands | y | y | y | / | y | / | y | / | y | y | y | y | / | same | / |
| Mature regenerated tail spotted | / | mottled | / | mottled | mottled | / | / | / | / | / | / | / | / | / | / |
| **Morphometrics** | | | | | | | | | | | | | | | |
| SVL | 68.5 | 67.7 | 67.7 | 74.4 | 69.2 | 60.4 | 59.6 | 67.0 | 61.8 | 33.2 | 35.1 | 32.0 | 30.9 | 36.5 | 34.7 |
| TL | 78.1r | 85.0r | 87.0 | 77.0r | 78.0r | 12.3b | 78.0 | 27.5r | 75.0 | 36.7 | 40.0 | 38.4 | / | 50.0 | 7.1b |
| TW | 6.9 | 6.4 | 6.3 | 7.0 | 5.5 | 6.3 | 5.1 | 8.0 | 6.2 | 2.8 | 3.0 | 2.8 | 2.6 | 3.2 | 4.0 |
| FL | 11.1 | 11.9 | 11.3 | 11.7 | 11.2 | 9.9 | 10.2 | 11.2 | 9.9 | 5.5 | 5.4 | 5.5 | 5.1 | 5.8 | 5.3 |
| TBL | 13.6 | 14.4 | 13.6 | 14.2 | 13.2 | 12.3 | 11.7 | 14.2 | 12.8 | 6.8 | 6.4 | 6.3 | 6.1 | 6.7 | 6.4 |
| AG | 31.5 | 30.0 | 29.7 | 32.9 | 29.8 | 26.9 | 26.7 | 30.1 | 26.3 | 11.9 | 14.1 | 13.6 | 12.0 | 16.3 | 13.9 |
| HL | 18.9 | 21.2 | 19.8 | 21.2 | 19.7 | 18.2 | 17.6 | 19.9 | 17.9 | 11.0 | 10.8 | 10.2 | 11.0 | 11.4 | 11.8 |
| HW | 13.6 | 14.2 | 13.5 | 14.7 | 13.3 | 12.4 | 12.0 | 13.6 | 12.6 | 7.5 | 7.6 | 6.4 | 6.7 | 7.9 | 7.6 |
| HD | 7.6 | 8.9 | 7.4 | 7.8 | 7.8 | 7.6 | 6.5 | 7.3 | 6.7 | 4.2 | 4.5 | 4.6 | 4.0 | 5.2 | 4.3 |
| ED | 4.4 | 4.6 | 4.3 | 4.8 | 4.4 | 3.6 | 4.1 | 4.4 | 3.9 | 2.9 | 2.7 | 3.0 | 2.7 | 2.9 | 2.8 |
| OD | 5.9 | 6.3 | 5.5 | 6.6 | 5.5 | 4.9 | 5.0 | 5.7 | 5.1 | 3.7 | 3.6 | 3.7 | 3.5 | 4.0 | 3.9 |
| EE | 5.1 | 5.8 | 5.5 | 5.9 | 5.6 | 5.0 | 4.9 | 5.3 | 5.4 | 2.6 | 3.1 | 2.3 | 2.8 | 2.9 | 2.8 |
| ES | 7.5 | 7.8 | 7.7 | 8.2 | 7.6 | 7.2 | 7.0 | 7.7 | 7.0 | 4.1 | 4.4 | 4.0 | 3.9 | 4.4 | 4.3 |
| EN | 5.6 | 5.8 | 5.7 | 6.1 | 5.7 | 5.1 | 5.1 | 5.9 | 5.1 | 2.8 | 2.9 | 2.8 | 3.0 | 3.2 | 2.9 |
| IO | 5.8 | 4.5 | 4.7 | 5.2 | 5.0 | 4.2 | 5.1 | 2.1 | 4.2 | 3.3 | 3.3 | 3.1 | 3.1 | 3.4 | 3.3 |
| EL | 2.0 | 2.2 | 2.1 | 2.4 | 2.1 | 1.5 | 2.0 | 1.8 | 2.0 | 1.2 | 1.0 | 1.3 | 1.1 | 1.3 | 1.1 |
| IN | 2.0 | 2.1 | 2.1 | 2.1 | 2.0 | 2.0 | 2.0 | 5.2 | 2.0 | 1.4 | 1.2 | 1.2 | 1.3 | 1.4 | 1.2 |
